# Automated Detection and Evaluation of Swallowing Using a Combined EMG/Bioimpedance Measurement System

**DOI:** 10.1155/2014/405471

**Published:** 2014-07-10

**Authors:** Corinna Schultheiss, Thomas Schauer, Holger Nahrstaedt, Rainer O. Seidl

**Affiliations:** ^1^Ear, Nose and Throat Unit, Unfallkrankenhaus Berlin, Warener Straße 7, 12683 Berlin, Germany; ^2^Control Systems Group, Technische Universität Berlin, Germany

## Abstract

*Introduction*. Developing an automated diagnostic and therapeutic instrument for treating swallowing disorders requires procedures able to reliably detect and evaluate a swallow. We tested a two-stage detection procedure based on a combined electromyography/bioimpedance (EMBI) measurement system. EMBI is able to detect swallows and distinguish them from similar movements in healthy test subjects. *Study Design*. The study was planned and conducted as a case-control study (EA 1/019/10, and EA1/160/09, EA1/161/09). *Method*. The study looked at differences in swallowing parameters in general and in the event of penetration during swallows in healthy subjects and in patients with an oropharyngeal swallowing disorder. A two-stage automated swallow detection procedure which used electromyography (EMG) and bioimpedance (BI) to reliably detect swallows was developed. *Results*. Statistically significant differences between healthy subjects and patients with a swallowing disorder were found in swallowing parameters previously used to distinguish between swallowing and head movements. Our two-stage algorithm was able to reliably detect swallows (sensitivity = 96.1%, specificity = 97.1%) on the basis of these differences. *Discussion*. Using a two-stage detection procedure, the EMBI measurement procedure is able to detect and evaluate swallows automatically and reliably. The two procedures (EMBI + swallow detection) could in future form the basis for automated diagnosis and treatment (stimulation) of swallowing disorders.

## 1. Introduction

For diagnosis and treatment of swallowing disorders we require a reliable identification and evaluation of swallowing. The imaging procedures used as golden standard (fiberoptic endoscopic evaluation on swallowing (FEES) and videofluoroscopy (VFSS)) are insufficiently reliable and are not often repeatable in case of the VFSS [1, 2]. For the use in the clinical everyday life new procedures become necessary which allow an objective swallowing detection and if necessary assessment. From this arise other applications like support swallowing by functional electrical stimulation.

Crucial for automated stimulation for therapeutic purposes or for a neuroprosthetic device is a measurement system able to detect and classify swallows. Swallow detection systems are used to identify swallows within a data set. Swallows are classified based on specific characteristics of a swallow identified by applying an automated classifier. To be able to trigger stimulation at the appropriate time point, both detection and classification need to occur at a stage as early as possible. Various measurement procedures, including electromyography (EMG) [3] and accelerometers [4], have been studied in relation to this issue. Both methods enable swallows to be detected on the basis of learning algorithms but are not able to reliably distinguish swallows from similar head and neck movements [4] or are only able to do so when applied to the same data sets as were used to train the algorithms [5]. For clinical use, especially in patients with complex swallowing disorders, any measurement system needs to deliver robust results and allow signals from similar movements, such as chewing and swallowing, to be distinguished reliably. This should be possible using surface electrodes.

Using a combination of EMG and bioimpedance signals (EMBI), Schultheiss et al. were able to present a measurement system meeting the above requirements [6]. The system is well tolerated and simple to use and delivers reproducible results. Their investigations demonstrated that the ability to differentiate between swallowing and head movements using swallowing-related parameters (e.g., speed of laryngeal elevation) extracted from these signals is statistically significant.

Diagnostic and therapeutic use of such a measurement system, as part of a neuroprosthetic swallowing device, for example, would require automated detection of swallowing and differentiation from similar head movements. This study aims to investigate the ability to detect and to describe the quality of swallows automatically.

## 2. Materials and Methods

### 2.1. Test Subjects

The study investigated healthy subjects with no swallowing disorder and patients with an oropharyngeal swallowing disorder. The patient group was planned as a mixed population (neurological and ENT specific disorders) for including a wide range of swallowing disorders. Exclusion criteria for all test subjects were pregnancy, a cardiac pacemaker, defibrillator, stent, or central venous catheter. All test subjects were given an explanation of the procedure and agreed to sign a consent form. The study was approved by the Charité Berlin Ethics Committee in votes EA 1/019/10, EA1/160/09, and EA1/161/09.

### 2.2. Measuring Device and Electrodes

**Figure 1:**
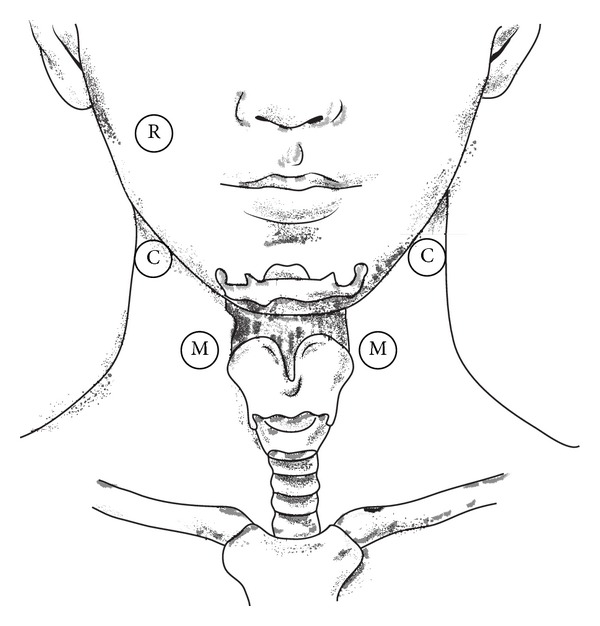
Electrode positioning. R = reference electrode, M = voltage measurement electrodes, and C = current source electrodes.

Measurements were made using a combined EMG/bioimpedance (EMBI) measurement system [7]. The EMBI system permits simultaneous measurement of bioimpedance (BI) and electromyography (EMG). Measurements were made using surface electrodes. The current electrodes were positioned bilaterally at the insertion of the sternocleidomastoid muscle. The measurement electrodes were attached bilaterally between the hyoid and larynx. The reference electrode was attached to the right cheek at the level of the cheekbone (see Figure 1). The number of electrodes used had previously been investigated during evaluation of EMBI [6]. The number of electrodes and the electrode positions were chosen based on the existing literature [8].

#### 2.2.1. Measured Signal

**Figure 2:**
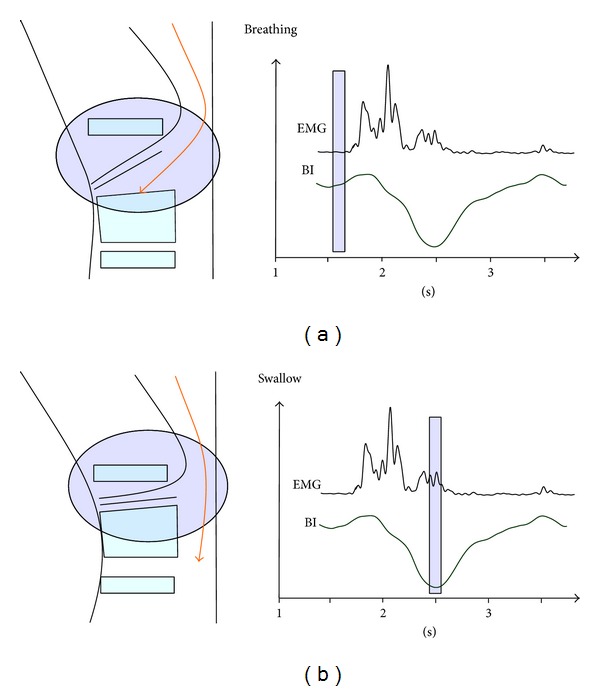
Schematic of bioimpedance measurement. (a) During breathing, the pharynx is filled with air and tissue resistance is consequently high. (b) On swallowing, the pharynx is closed through muscle action and resistance is reduced.

Bioimpedance is a complex resistance parameter defined as the ratio of voltage to current arising across an electrical conductor. The ratio of voltage to current in the pharyngeal cavity changes depending on activity (swallowing/breathing, Figure 2). During breathing, the pharyngeal cavity is open and filled with air. Since air is a poor conductor, its electrical resistance is high. During a swallow, the pharyngeal cavity narrows as a result of the upward/forward movement of the hyoid and larynx. Electrical resistance falls, as the space is filled with tissue. Tissue is a good electrical conductor. The deviation of BI was detected with piecewise linear approximation (PLA) [9]. The PLA detects valleys in the BI signal. These valleys have to fulfill several conditions and enable calculating the swallowing-related start and end points in the BI signal [10].

EMG measures the electrical activity of striated muscles. In this study, we measured the EMG of the submental muscle group, which is responsible for elevating the larynx and hyoid. EMG activity during swallowing always commences prior to the drop in BI. The start and end of EMG activity were detected by using a double-threshold detector [11] on the high pass filtered EMG signal with 4 kHz [10].

**Figure 3:**
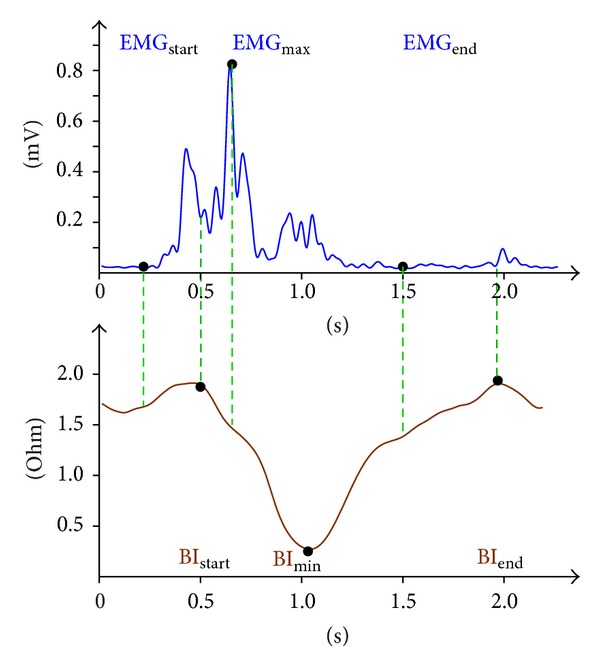
Biosignal curve. The signal trace above shows typical EMG (mV) and BI (ohm) biosignal curves for a saliva swallow in a healthy subject. BI_start_ = start of laryngeal elevation, BI_min⁡_ = maximum laryngeal elevation (BI_min⁡_ − BI_start_), BI_end_ = end of laryngeal elevation, EMG_start_ = start of muscle activity, EMG_max⁡_ = maximum muscle activity during laryngeal elevation, and EMG_end_ = end of muscle activity. Other swallowing-related parameters are speed of laryngeal elevation ((BI_min⁡_ − BI_start_)/(*t*(BI_min⁡_) − *t*(BI_start_))), extent of laryngeal elevation (BI_start_ − (*t*(BI_min⁡_) − *t*(BI_start_))), and duration of preparation for swallowing and bolus formation (*t*(BI_start_) − *t*(EMG_start_)).

The biosignal (BI and EMG) curves during swallowing have characteristic shapes (see Figure 3) which correlate with the pharyngeal phase of swallowing [6]. The biosignal parameters listed can be used to derive or calculate swallowing-related parameters which describe the swallowing process. These parameters are required in order to perform a statistical comparison between healthy subjects and patients with a swallowing disorder [6]. “Duration of preparation for swallowing and bolus formation,” for example, is observed retrospectively commencing with the start of the drop in BI, giving rise to a negative figure. “Maximum laryngeal elevation” and “speed of laryngeal elevation,” the latter calculated from the former, also prove to be negative, since the drop in BI is measured starting from zero. Both parameters have an important role to play in distinguishing swallows in healthy subjects from swallows in patients with a swallowing disorder.

#### 2.2.2. Detecting Swallowing

The EMG and bioimpedance signals were filtered and the noise overlaying the signals was removed. A two-stage procedure based on detecting and classifying physiological criteria during swallowing was used for automated swallow detection. Identification of EMG activity represented a first filter criterion and was used to segment the biosignal, in accordance with the rule that swallowing is not possible without muscle and thus EMG activity. Identification of falls in BI in the segments with EMG activity was used as a second selection criterion. This involved identifying troughs using linear approximation. A region with EMG activity and a drop in BI thus represent a potential swallow. The next step in differentiating swallows from nonswallows was to apply a classifier which had been trained to detect swallows on a comparison group [10]. A combination of physiological criteria and classifiers was required, as neither procedure delivered satisfactory results individually. Identifying swallows based on EMG activity (physiological criterion) alone was insufficient, as it did not permit swallows to be differentiated from other movements, such as head movements. As a reference point for data analysis, all swallows in our study data set were marked out by the author (C.Sch.) by hand. Subjects were asked to hold a bolus in the oral cavity before swallowing and to indicate swallows during the course of our investigations by pressing a button.

### 2.3. Procedure for Investigations

The data from healthy subjects used here is taken from our previous study evaluating EMBI [6]. This evaluation involved asking subjects to swallow various volumes (5 mL, 10 mL, and 20 mL) and consistencies (saliva, liquid, semisolid, and solid).

Patients were investigated by a doctor and a speech therapist in a swallowing disorder clinic. Patients were seated upright in a chair and placed the food in their mouths themselves. Findings were evaluated by both investigators. None of the patients experienced any side effects as a result of the methods used.

EMBI measurement was carried out in parallel with fiberoptic endoscopic evaluation of swallowing (FEES). In preparing for the investigation, electrodes were placed as shown in Figure 1. Cables were used to connect the electrodes to the measuring equipment, which was in turn connected to the USB port of a laptop, which recorded the data produced.

In addition to technical preparations, the foods to be used for the investigations were also provided. Saliva and three consistencies of food, still water containing blue food colouring, green jelly, and small pieces of bread with the crusts removed and spread with a spread, were tested using FEES. Endoscopic evaluation of swallowing was performed by a physician using the Berlin Dysphagia Index (BDI, mean score = 7 (therapy necessary)) [12].

### 2.4. Statistical Analysis

Statistical analysis was carried out using the software SPSS 20. Sensitivity and specificity (the criteria for calculating the efficiency of automatic swallow detection) were calculated using a 2 × 2 contingency table. Comparisons between healthy subjects and patients with a swallowing disorder were performed with the aid of the Mann *U* test. Comparisons within the patient group (swallows with and without penetration) were also performed with the aid of the Mann *U* test. A significance level *P* ≤ 0.05 provided adequate evidence that there was a statistically significant difference between the comparison groups.

## 3. Results

### 3.1. Test Subjects

Over a two-year period we investigated 31 healthy subjects (♀ = 15, ♂ = 16) and 41 patients (24 neurological and 17 ENT specific) with a swallowing disorder (♀ = 15, ♂ = 26). The age of the healthy subjects ranged from 24.0 to 51.0 (the mean age was 32.5 ± 7.8). The age of the patients with a swallowing disorder ranged from 24.0 to 93.0 (mean age was 63.4 ± 13.8) (see Table 1). Patients were evaluated using FEES and were shown to have an oropharyngeal swallowing disorder.

### 3.2. Measured Signal/Swallows

A total of 1828 swallows by healthy subjects and 711 swallows by patients with a swallowing disorder were recorded (see Table 2). Healthy subjects were tested as part of the process of evaluating EMBI and were involved in multiple studies (distinguishing between swallowing and head movements and the effects of different volumes and consistencies on the EMG and BI signals). EMBI was evaluated using healthy subjects as it was not possible to perform such a large number of swallows on patients with a swallowing disorder and we wished to keep the stress placed on such patients to a minimum. The presence of a swallowing disorder limited the number of swallows by patients with a swallowing disorder which could be observed. Swallowing tests were evaluated on the basis of video recordings of endoscopies, which were used to determine whether any penetration was present. This information was then used to train the classifier to detect swallows automatically. Swallowing-related parameters were determined for each swallow for each test subject (Figure 3). Statistical comparisons were undertaken primarily on the basis of parameters recorded in the literature as being crucial for safe swallowing, specifically maximum laryngeal elevation and speed of laryngeal elevation [13, 14]. In addition, bolus formation and preparation for swallowing were used in evaluating swallows by patients with a swallowing disorder.

### 3.3. Comparison of Swallowing Parameters

Swallowing-related EMBI parameters extracted from the EMBI measurement curves were compared between the two groups (healthy subjects and patients with a swallowing disorder). The Mann *U* test found a statistically significant difference between swallows in healthy subjects and swallows in patients with swallowing disorders for all swallowing-related parameters. Healthy subjects had a mean swallowing preparation time of −0.2 seconds (saliva = −0.3 s, liquid = −0.2 s, semisolid = −0.2 s, and solid = −0.03 s) and a maximum laryngeal elevation of −1.4 ohm (saliva = −1.5 ohm, liquid = −1.3 ohm, semisolid = −1.6 ohm, and solid = −1.8 ohm) at a speed of −5.0 ohm/s (saliva = −4.8 ohm/s, liquid = −4.7 ohm/s, semisolid = −5.8 ohm/s, and solid = −5.1 ohm/s). Patients with swallowing disorders had a significantly shorter overall swallowing preparation time (overall = 0.02 s, saliva = −0.2 s, liquid = −1.1 s, semisolid = −0.8 s, and solid = 0.06 s), smaller magnitude of laryngeal elevation (overall = −1.2 ohm, saliva = −1.2 ohm, liquid = −1.2 ohm, semisolid = −1.1 ohm, and solid = −1.2 ohm), and slower laryngeal elevation (overall = −4.3 ohm/s, saliva = −4.3 ohm/s, liquid = −4.4 ohm/s, semisolid = −4.2 ohm/s, and solid = −3.9 ohm/s) (see Table 3).

### 3.4. Swallowing Parameters and Penetration

664 of 711 swallows were able to be classified on the basis of endoscopic imaging. 47 swallows were not able to be classified due to defective data or poor quality endoscopy images. A total of 587 swallows with no penetration and 77 swallows involving penetration were observed. Statistically significant difference in the swallow-related parameters described above was not found for the two groups of patients (with penetration or with no penetration; see Table 4). Swallows in which penetration did not occur showed a smaller overall maximum laryngeal elevation (overall = −1.2 ohm, saliva = −1.1 ohm, liquid = −1.2 ohm, semisolid = −1.1 ohm, and solid = −1.3 ohm) than swallows involving penetration (overall = −1.3 ohm, saliva = −1.3 ohm, liquid = −1.8 ohm, semisolid = −1.2 ohm, and solid = −2.1 ohm). Speed of laryngeal elevation was smaller in swallows in which penetration did not occur (overall = −4.2 ohm/s, saliva = −4.1 ohm/s, liquid = −4.3 ohm/s, semisolid = −4.4 ohm/s, and solid = −4.0 ohm/s) than in swallows involving penetration (overall = −4.8 ohm/s, saliva = −5.0 ohm/s, liquid = −6.5 ohm/s, semisolid = −3.8 ohm/s, and solid = −7.8 ohm/s).

### 3.5. Automatic Swallow Detection

EMBI measurement data from nine healthy subjects (♀ = 2, ♂ = 7, 1360 swallows) was used to train and test automated swallow detection.

The first step involved segmenting the EMBI signal; that is, the swallowing-specific drop in BI (2nd selection criterion) had to coincide with a muscular action (1st selection criterion), visible as EMG activity. This enabled 99.3% of swallows to be correctly detected. 4128 nonswallows were also wrongly identified as swallows (false positives). The second step involved using a classifier (support vector machine [15]) to improve differentiation. A classifier is trained to recognise typical characteristics of a swallow using a known data set with characteristic details. A total of 1360 swallows and 4128 nonswallows were captured for use in training (subjects 1–5) and testing (subjects 6–9) the classifier. 698 swallows and 2433 nonswallows were used to train the classifier. Data from 662 swallows and 1695 nonswallows, selected on the basis of physiological criteria, was then tested for the occurrence of swallows using the trained classifier. This combined procedure was able to achieve a sensitivity of 96.1% and a specificity of 97.1% [10], yielding an accuracy of 96.6%.

In order to apply the automated swallow detection procedure to patients with swallowing disorders, a total of 711 swallows and 1869 nonswallows from 41 patients were divided between training and test data sets. 284 swallows and 1264 nonswallows (20 patients) were assigned to the training data set and 427 swallows and 1527 nonswallows (21 patients) to the test data set. The sensitivity of automated swallow detection was 84.1% and specificity 84.7%. Accuracy of automated swallow detection was 84.5%.

## 4. Discussion

At present, clinical diagnosis of swallowing disorders is based on evaluation by an investigator and is consequently solely dependent on the experience of that investigator. This dependency is illustrated by the relatively low figures for interrater reliability of 40–69% for videofluoroscopy [2] and 51% for video endoscopy [1].

In a previous paper we presented a noninvasive combined EMG/bioimpedance (EMBI) measurement system for evaluating swallowing [6]. This system allows functional changes in the pharynx during swallowing to be modelled with a very high degree of reproducibility. The mean correlation coefficient for intrarater reliability $\overset{-}{r}$ was 0.878–0.992 (over 80%) and the mean intraclass correlation coefficient for interrater reliability $\overset{-}{\text{ICC }}$ was 0.846 (over 80%).

In contrast to healthy test subjects, patients with a swallowing disorder exhibit changes to their swallow. The progression of hyoid and laryngeal movement is slower in patients with aspiration. Kendall et al. describe pharyngeal dysphagia as the interplay between reduced hyolaryngeal elevation, reduced pharyngeal contraction, and reduced opening of the pharyngoesophageal segment. Drawing on this, one study found a reduced duration of maximum laryngeal elevation and an increased duration of the swallow as a whole [16].

Studies on automated classification of swallowing have been carried out based on procedures including electromyography and accelerometry. By detecting swallowing based on a combination of surface electromyography (sEMG), swallow apnoea, and cervical auscultation, Crary et al. were able to demonstrate a sensitivity and specificity of more than 90% [3]. Work on accelerometer-based automated detection of swallowing has used a range of different classifiers. Sazonov et al. described the use of a support vector machine (SVM) [17]. They used swallowing sounds to achieve a detection accuracy of more than 80%. As long ago as 2004, Aboofazeli and Moussavi described the use of a neural network, which achieved an accuracy of more than 90% [18]. Both studies examined swallow detection in healthy subjects only.

In our study, we developed an algorithm which is able to reliably detect swallows using two measurements (EMG and BI). We started from the observation that swallowing is always the consequence of muscle activity and is instituted by an EMG change. This applies equally to many other orofacial activities, however, for example, chewing. Consequently, a second procedure able to evaluate changes in the bioimpedance curve and able to utilise small changes in the shape of the curve was required. With the help of a support vector machine, it proved possible to reliably distinguish a swallow from other movements (chewing). This two-stage procedure is necessary, as use of a single detection procedure alone produces a large number of false positives.

The sensitivity and specificity of automated differentiation between swallow and head movements using the procedure presented here were very good (both over 90%) and its accuracy was 96.6%. In patients with a swallowing disorder, the sensitivity of automated swallow detection was 84.1%, specificity was 84.7%, and accuracy was 84.5%. Expanding the base data will in future enable the system presented here to detect swallows automatically and enable swallowing disorders to be investigated more reliably.

## 5. Summary

The EMG/bioimpedance measurement system presented here is able to map characteristic functional changes in the pharynx during swallowing. Differences in functional changes (laryngeal elevation, etc.) in patients with swallowing disorders form the basis for a procedure for detecting and evaluating swallows automatically, which has been implemented as a two-stage procedure. This lays the groundwork for automated evaluation of the swallowing process for diagnostic purposes and for therapeutic stimulation of swallowing using internal or external stimulators.

## Figures and Tables

**Table 1:** Summary of test subjects.

Investigation	Number	Age	Medical history
Control	31	32.5 ± 7.8	Healthy
FEES	41	63.4 ± 13.8	Swallowing disorder

24 of the 41 patients had neurological disorders: 10/24 = neurodegenerative disease, 8/24 = stroke, 2/24 = heart attack, 2/24 = craniocerebral injury, and 2/24 = other. 17 of the 41 patients had ENT specific tumor diseases.

**Table 2:** Summary of swallows.

Group	Number	Medical history	Swallow detection
Test subjects	1828	Healthy	Training = 1360
			Test = 698

Patients	711	wnP = 587	Training = 284
		wP = 77	Test = 427

wnP = with no penetration, wP = with penetration, and number = number of swallows.

**Table 3:** Group swallow parameters.

Characteristic	Medical history	Mean	SD	Statistical significance
Duration of preparation for swallowing and bolus formation	Healthy	−0.192	0.512	0.000∗∗∗
	Swallowing disorder	0.022	8.360	

Extent of laryngeal closure	Healthy	−0.935	0.512	0.013∗
	Swallowing disorder	−0.955	0.219	

Maximum laryngeal elevation	Healthy	−1.416	0.482	0.000∗∗∗
	Swallowing disorder	−1.170	0.645	

Speed of laryngeal elevation	Healthy	−4.963	1.901	0.000∗∗∗
	Swallowing disorder	−4.253	2.488	

The comparison above shows means and standard deviations (SD) for both healthy subjects and patients with a swallowing disorder. The significance level was calculated using the Mann *U* test, where ∗∗∗ denotes a statistically significant difference at a significance level of 0.01 and ∗ at a significance level of 0.05.

**Table 4:** Patient swallowing parameters.

Characteristic	Medical history	Mean	SD	Statistical significance
Duration of preparation for swallowing and bolus formation	wnP	0.029	9.172	0.352
	wP	−0.059	1.600	

Extent of laryngeal closure	wnP	−0.954	0.217	0.370
	wP	−0.928	0.210	

Maximum laryngeal elevation	wnP	−1.157	0.621	0.495
	wP	−1.332	0.848	

Speed of laryngeal elevation	wnP	−4.758	3.416	0.710
	wP	−4.964	1.901	

The comparison above shows means and standard deviations (SD) for patients with a swallowing disorder. The significance level was calculated using the Mann *U* test, where ∗∗∗ denotes a statistically significant difference at a significance level of 0.01 and ∗ at a significance level of 0.05. wnP = with no penetration and wP = with penetration.
